# Banded Sleeve Gastrectomy Improves Weight Loss Compared to Nonbanded Sleeve: Midterm Results from a Prospective Randomized Study

**DOI:** 10.1155/2020/9792518

**Published:** 2020-05-28

**Authors:** Paolo Gentileschi, Emanuela Bianciardi, Leandro Siragusa, Valeria Tognoni, Domenico Benavoli, Stefano D'Ugo

**Affiliations:** ^1^Department of Surgery, Obesity Unit, Tor Vergata University Hospital, Viale Oxford 81–00133, Rome, Italy; ^2^Department of Systems Medicine, Tor Vergata University Hospital, Viale Oxford 81–00133, Rome, Italy; ^3^Department of Surgery, Tor Vergata University Hospital, Viale Oxford 81–00133, Rome, Italy; ^4^Department of General Surgery, “Vito Fazzi” Hospital, Piazza Muratore 1–73100, Lecce, Italy

## Abstract

**Background:**

Weight regain after laparoscopic sleeve gastrectomy (LSG) is nowadays a growing concern. Sleeve dilatation and loss of food restriction is considered the main mechanism. The placement of a silicon ring around the gastric tube seems to give benefits in the short term. We report the results of a randomized study comparing LSG and laparoscopic banded sleeve gastrectomy (LBSG) over a 4-year follow-up.

**Objectives:**

To evaluate the efficacy of banded sleeve gastrectomy compared to standard sleeve in the midterm.

**Methods:**

Between 01/2014 and 01/2015, we randomly assigned 50 patients to receive one of the two procedures. Patients' management was exactly the same, apart from the band placement. We analyzed differences in weight loss, operative time, complication rate, and mortality, with a median follow-up of 4 years.

**Results:**

Twenty five patients were assigned to receive LSG (Group A) and 25 LBSG (Group B). The mean preoperative BMI (body mass index) was 47.3 ± 6.58 kg/m^2^ and 45.95 ± 5.85 kg/m^2^, respectively. There was no significant difference in the operative time. No intraoperative or postoperative complications occurred. At 12-month follow-up, the mean BMI was 29.72 ± 4.40 kg/m^2^ in Group A and 27.42 ± 4.47 kg/m^2^ in Group B (*p*=0.186). After a median follow-up of 4 years, the mean BMI in Group B was significantly lower than Group A (24.10 ± 4.52 kg/m^2^ vs 28.80 ± 4.62 kg/m^2^; *p*=0.00199).

**Conclusions:**

LBSG is a safe procedure, with no impact on postoperative complications. The banded sleeve showed a significant greater weight loss in the midterm follow-up. Considering the issue of weight regain observed after LSG, the placement of a perigastric ring during the first procedure may be a strategy to improve the results. This trial is registered with NCT04228185.

## 1. Introduction

Laparoscopic sleeve gastrectomy (LSG) with its growing popularity has nowadays become the most performed bariatric procedure worldwide [1, 2]. Originally part of a first-stage approach in superobese patients, LSG is now widely accepted as a stand-alone procedure [3, 4]. This is due to its safety and effectiveness, technical simplicity, short learning curve, reduced operative time, feasibility even in super-superobese patients, and chances of revision and conversion to malabsorptive surgery [5, 6]. Furthermore, data already demonstrated a comparable midterm weight loss with LRYGB and no significant difference in the resolution of obesity-related comorbidities [7–9].

Despite the evident pros of LSG, LRYGB still remains the gold standard procedure. The main concern is the long-term weight regain after LSG related to the purely restrictive mechanism [10]. Reasons of insufficient weight loss and weight regain are multifactorial, and factors leading to the LSG failure in weight control are still controversial [11–13]. However, an increase in the size of the gastric reservoir due to its dilation over time is suggested to be the leading cause of this phenomenon, and that it is the underlying rationale behind the indication to resleeve gastrectomy [14–16].

There is evidence of weight loss flattening out and initial weight regain starting from 2 years after surgery [17]. Redoing surgery after LSG is a challenge, and the rate of recurrence and inadequate weigh loss is significant; some centers report that up to 30% of patients need revisional surgery [18].

Efforts in avoiding weight regain brought, at first, bariatric surgeons to band the gastric pouch of LRYGB in order to prevent gastric dilation and eventually improve the weight loss maintenance [19]. Weight loss related to the band placement is considered to be a mix of malabsorptive and restrictive mechanism: reduced food intake, effect on endoluminal pressure and esophageal peristalsis, hormonal effect, and altered gastric emptying [20].

As for LRYGB, surgeons applied the same principle to LSG, placing an adjustable device around the sleeve pouch just below the gastroesophageal junction [21].

Hereby, we present the first randomized control trial in the literature comparing standard LSG and BSLG, using the GaBP Ring Autolock, with a 4-year follow-up.

## 2. Materials and Methods

**Figure 1:**
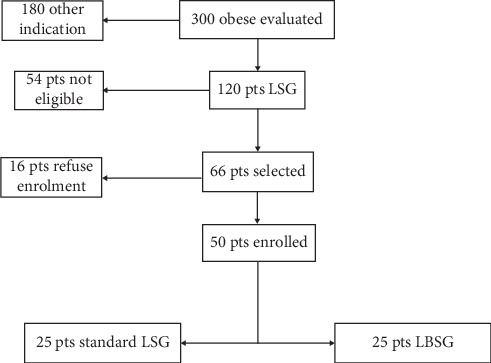
Flow diagram of patients' selection.

The study analyzed data of a prospective randomized trial from a single referral bariatric center, after a median follow-up of 4 years. The selection of patients to be included in the study was performed in 2013 with a total of 300 bariatric patients screened. Out of these, 120 patients were selected for LSG. Exclusion criteria were age below 18 years or over 60, previous bariatric or gastrointestinal surgery, psychiatric contraindications, pregnancy, and other medical reasons for denying laparoscopy. According to those, 54 patients were excluded from the study; of the remaining 66, 16 refused to be included in the protocol. Patients enrolled were randomly assigned into 2 samples: Group A, including 25 obese patients undergoing standard LSG, and Group B, including 25 obese patients undergoing banded LSG (Figure 1). The randomization was obtained by drawing two opaque envelopes containing, respectively, a card with the indication to LSG or LBSG. A power analysis, based on the main endpoint, was performed by a statistician.

All the patients were invited to participate to this study and were informed in detail about the risks and the benefits of each procedure; all the participants included in the study gave their written informed consent.

All the procedures involving human participants were done in accordance with the ethical standards of the institutional and/or national research committee and with the 1964 Helsinki Declaration and its later amendments or comparable ethical standards. The study was approved by the local ethic committee.

### 2.1. Preoperative Management

A multidisciplinary team evaluated the candidates based on medical, nutritional, endocrinological, and psychiatric workup. The standard preoperative assessment included barium X-ray of the upper gastrointestinal tract and esophagogastroduodenoscopy, blood examinations, cardiology evaluation, and chest radiography. Psychiatric counselling was conducted to evaluate mental health contraindications to surgery. A semistructured interview was performed with the aim of exploring patients' weight and dieting history, motivation for seeking surgery, and expectations concerning the surgical outcome [22].

### 2.2. Surgical Technique

All the procedures were performed laparoscopically, using four or five ports, by the same surgeon (PG). LSG was performed with 36-F bougie, and the gastric resection was carried out with a reinforced linear stapler. The ring used was the GaBP Ring Autolock System, composed of a radiopaque silicon-coated implantable device with a plastic one-way lock mechanism at the ends of the ring. It was placed 4 cm distal from the cardia hiatus through a retrogastric tunnel created in the pars flaccida of the hepatogastric ligament. The diameter of the ring selected to be used in our study was 7 cm; only two patients received a 7.5 cm ring due to excessive narrowing of the gastric tube. To prevent any displacement of the ring, we used two loose nonresorbable stitches.

### 2.3. Postoperative Management

Every patient underwent an upper gastrointestinal swallow test with Gastrografin on the second postoperative day. Alimentary advices included a diet consisting of clear liquids and pureed foods for 15 days and a semisolid diet for the next 15 days. After the first 30 days, patients gradually began a low-fat, low-carbohydrate, high-protein solid diet based on the advice of a dietitian.

### 2.4. Data Analysis

Preoperative patients' data such as BMI (body mass index), obesity-related comorbidities (hypertension, T2 diabetes mellitus (T2DM), and obstructive sleep apnea syndrome (OSAS)), and home therapy were included in our prospective database. Intra- and postoperative data included operative time, adverse events or complications, and hospital stay. After discharge, follow-up appointments were at 15 days and then 1, 3, 6, 12, 36, and 48 months after surgery.

Intolerance to solid food and remission of hypertension as well as T2DM or OSAS have been evaluated during each follow-up. Remission of hypertension and T2DM has been considered for values of pressure <140/80 mmHg and glycemia <126 gr/dL, after suspension of medications for at least 1 year. Resolution of OSAS was considered stable in patients able to stop using C-PAP or with significant clinical improvement for minimum 12 months.

The primary outcome of the study was to assess the weight loss between the two groups in the midterm, analyzing data after a median follow-up of 4 years. Secondary endpoints were the evaluation of complications and resolution of obesity-related comorbidities.

Statistical analyses were performed using IBM SPSS version 20 for Windows. Categorical variables were analyzed using the chi-squared test and Fisher's exact test. Interaction between surgical treatment and weight loss over time was assessed with the ANOVA test. *p* values are two sided, and values < 0.05 were considered statistically significant.

## 3. Results

Of the fifty obese patients enrolled in the study, 25 were randomly assigned to Group A-LSG (16 females; 9 males) and 25 to Group B-LBSG (14 females; 11 males).

In Group A, the mean age was 43.7 ± 9.8 years and the mean preoperative BMI was 47.3 ± 6.58 kg/m^2^; in Group B, 45.7 ± 12.7 years and 45.95 ± 5.85 kg/m^2^. Twelve patients had preoperative T2DM, 7 (28%) in Group A and 5 (20%) in Group B. Twenty one subjects were on antihypertensive medications, 14 (56%) in Group A and 7 (28%) in Group B. Eight patients had a diagnosis of OSAS, 6 (24%) in Group A and 2 (8%) in Group B. Preoperative data are summarized in Table 1.

Operative and early postoperative features were part of our preliminary study [23].

One patient developed gastric stenosis following LSG. He experienced food intolerance after restarting solid diet. An esophagogastroduodenoscopy confirmed the stenosis, which was located at the level of the angulus and not related to the ring. For this reason, the sleeve was converted to RYGB.

All the 49 patients had a follow-up of 48 months, 24 in Group A and 25 in Group B. We had no patients lost to follow-up.

The mean BMIs registered after 3, 6, and 12 months in Groups A and B were, respectively, 37.86 ± 5.72 kg/m^2^ and 37.58 ± 6.21 kg/m^2^ (*p* = ns); 33.64 ± 6.08 kg/m^2^ and 32.03 ± 5.24 kg/m^2^ (*p* = ns); 29.72 ± 4.40 kg/m^2^ and 27.42 ± 4.47 kg/m^2^ (*p* = ns). No statistically significant difference was found between the two groups.

**Figure 2:**
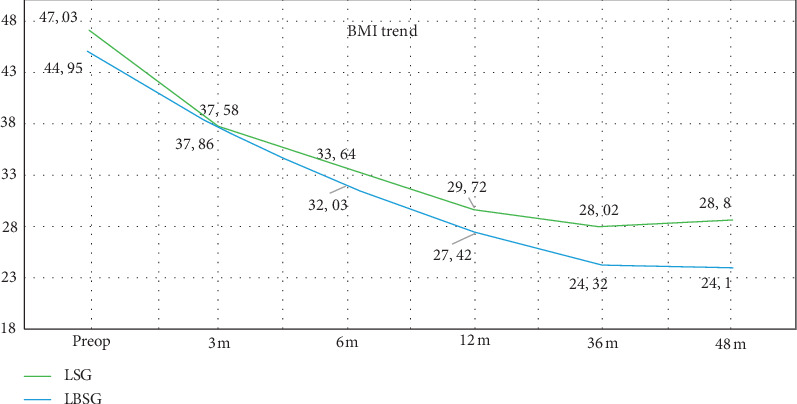
Variation of mean BMI during the follow-up. LSG: laparoscopic sleeve gastrectomy; LBSG: laparoscopic banded sleeve gastrectomy.

At 36-month follow-up, the mean BMI was 28.02 ± 4.21 kg/m2 in Group A and 24.32 ± 4.54 kg/m2 in Group B (*p* = 0.000205). At the end of 4-year follow-up, the mean BMI was 28.80 ± 4.62 kg/m^2^ in Group A and 24.10 ± 4.52 kg/m^2^ in Group B (*p* = 0.00199) (Table 2, Figure 2).

**Figure 3:**
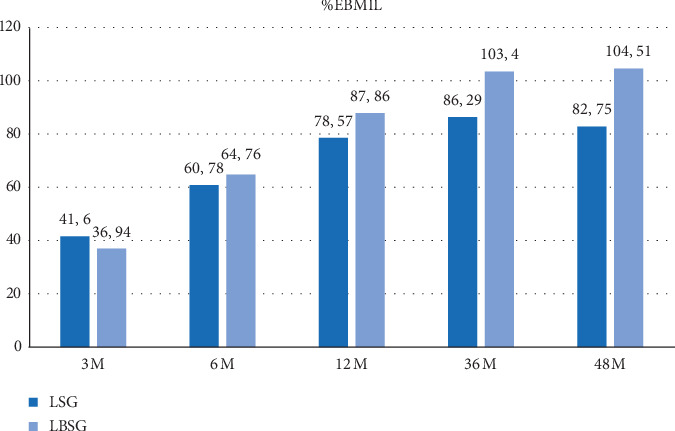
Variation of %EBMIL during the follow-up. LSG: laparoscopic sleeve gastrectomy; LBSG: laparoscopic banded sleeve gastrectomy; %EBMIL: % excess body mass index loss.

The %EBMIL (excess body mass index loss) at 36 months and 48 months was, respectively, 86.29% and 82.75% in Group A and 103.4% and 104.51% in Group B (*p* < 0.0001) (Figure 3).

In both groups, we had excellent results in terms of resolution of comorbidities after 4 years. Six patients in Group A (86% of diabetic subjects) and 4 in Group B (80%) had a complete resolution of T2DM after 6 months (*p*=0.755). Hypertension in the two groups decreased from 56% to 28% of patients in Group A and from 28% to 4% in Group B (*p*=0.022). After 6 months from surgery, no patients suffered from OSAS. These results remained stable after 1 year from surgery.

## 4. Discussion

Regardless of the bariatric procedure performed, the weight regain in the long term occurs in a small but significant number of patients, representing an important issue both for surgeons and patients [24].

The prevalence of weight regain has been reported to be higher in the restrictive techniques like LSG due to the dilatation of the gastric pouch in the long term [11, 12, 14–16]. Himpens et al. reported an excess weight loss (EWL) which decreased to 53% after 6 years, with the weight regain starting after 3 years from surgery [11]. Similarly, Alverenga et at. showed an EWL of 52% at 8 years from a large group of patients [25].

This topic led to the development of strategies and tools in order to prevent the weight regain after bariatric surgery. The use of a band or ring around the stomach was reported initially during the gastric bypass, with promising results; more recently, this concept has been extended to LSG [26, 27]. Among the first reports on LBSG, Alexander et al. used a band of biological tissue below the gastroesophageal junction. Comparing the weight loss with patients following banded RYGB, they found similar results after short-term follow-up [21].

Further studies were performed using a silicon-coated device; this is easy to handle inside the abdomen, flexible, and without risk of integration with the gastric wall. The ring is placed a few centimeters below the cardia, after creating a tunnel behind the gastric tube, opening the pars flaccida of the hepatogastric ligament. The band is then locked loosely around the sleeve, without any compression on the stomach. The dissection along the lesser curvature is minimal, with no risk of vascular damage.

The main benefit of the ring should start in the mid- and long-term from surgery, when the dilatation of the gastric pouch is responsible of the weight regain. However, previous studies on the effect of LapBand on weight loss showed that its mechanism is not a mere restriction of the ingested food, but relies significantly on the early satiety [20]. Hence, the LBSG could benefit of a double way to reduce the excess weight, both restrictive and endocrine.

Despite the large numbers of LSG performed around the world and the promising results with RYGB, the use of banding devices is still performed only in a few highly specialized centers. Furthermore, national registers still do not take LBSG into account as a standard operation, so that the exact number of procedures is unknown.

A few years ago, our group designed a pilot randomized trial including 50 obese patients, comparing early results of banded and not banded LSG, using the GaBP Ring Autolock System. In terms of operative data, LBSG was a safe and feasible procedure, with no differences in surgical times and complications. In the short-term follow-up, the study did not show any significant improvement of weight loss for the banded sleeve, even if the LBSG group had a mean BMI slightly lower than that of the control group [23].

In a systematic review, Parmar et al. collected data on all the published papers about LBSG, including the analysis 236 patients. The median follow-up was 1 year. Also considering these global numbers of procedures, the authors did not find any statistical improvement in the weight loss for the LBSG in the short-term follow-up [28]. The only study with long-term follow-up included in this review showed better results for LBSG, but with a too small number of patients (*n* = 10) to draw any conclusion [26].

If we consider that the main mechanism of action of the ring is to avoid the gastric pouch dilatation in the long term, it is not surprising that we face with the above results in the early phase after LBSG.

In this paper, we reported the results of our prospective randomized trial after completing a median follow-up of 4 years. Starting from 36 months from surgery, the mean difference in BMI became significant between the two study groups. The presence of the ring improved significantly the results of the bariatric procedure. The magnitude of this data is also confirmed when we considered the %EBMIL, exceeding the expected results in patients treated with LBSG. Based on this data, it appears that the improved weight loss persists even at the longer follow-up of 48 months, thus confirming that the main mechanism of the banding is the prevention of weight regain due to the gastric dilatation. In particular, patients after standard LSG showed a reduction of %EBMIL after 4 years, associated to a slight increase in the mean BMI, likely expression of the greater sleeve volume. On the other side, we observed exactly an opposite picture following LBSG.

Because of the still limited number of series reporting information about banded sleeve, literature data about its long-term effect are very scant. Apart from the abovementioned few patients in the study by Lemmens et al. [26], the only other paper which has been recently published is by Fink et al. In this retrospective matched-pair analysis, the authors reported the results of 51 patients undergoing LBSG after 5-year follow-up. The weight loss was compared to the one obtained following standard LSG; similarly to our data, they report a significant improvement in the LBSG group starting from 36 months from surgery, maintained after the following two years. Again, the main hypothesis postulated by the author was the prevention of the sleeve dilatation thanks to the perigastric ring [29].

One of the main concerns about the banding device is the risk of displacement, erosion, or slippage. This idea is based on the previous data reported with the use of adjustable LapBand, when many patients required a reoperation in order to sort out the complication [30]. Anyway, the two systems are completely different from each other. The adjustable band is wider than the ring and above all is applied tightening it around the stomach; this results in a constant and even increased pressure on the gastric wall over time, with repeated insufflations. The small and thin ring used in LBSG is left loose without any pressure on the sleeve; moreover, the almost nil dissection on the lesser omentum makes negligible the risk of displacement or slippage.

At present, the number of LBSG is too small, and the follow-up not long enough to make any definitive comment. However, based on a meta-analysis including more than 8000 patients after banded RYGB and with 10-year follow-up, the rate of those complications is very low (2.3% of erosion; 1.5% of slippage) [31].

In our previous report on the short-term results of this randomized trial, there was no statistical difference in complications between LBSG and LSG, and the banded technique achieved the same results about improvement of comorbidities. Also after the extended follow-up, we did not record any complication band-related and the pre-existing diseases kept improving over time. These results are in line with another long-term study, where only one case of slippage has been reported [29].

Despite being a prospective randomized trial, with all the patients fulfilling a 4-year follow-up, this study has some limitations. The sample size is modest, and this could underestimate the incidence of band-related complications. Aim of this trial was to test a possible improvement in the weight loss due to the ring; we have not made a systematic assessment of postoperative reflux or regurgitation. This topic seems to be a possible issue in LBSG, and it needs to be investigated appropriately in the future. Moreover, to confirm the ability of the ring in the prevention of gastric pouch dilatation, a CT-volumetry of the sleeve should be performed during the follow-up. Finally, we did not examine the possible influence of maladaptive eating patterns and psychiatric comorbidities that might be described in further studies [32].

## 5. Conclusions

LBSG is a feasible procedure with a short learning curve, with no impact on postoperative complications. The banded sleeve seems to be significantly more effective than the standard LSG in terms of weight loss in the midterm follow-up. These encouraging results should open the way to standardize LBSG as a defined procedure in the bariatric community, maybe lowering the need of revisional surgery.

Additional prospective studies with larger sample size and adequate follow-up are needed to trace any definitive conclusion.

## Figures and Tables

**Table 1:** Preoperative data.

Parameters	Group A (LSG *n* = 25)	Group B (LBSG *n* = 25)	Total (*n* = 50)	*P* (0.05)
Age (mean, SD)	43.7 ± 9.8	47.3 ± 6.58	45.5 ± 8.19	NS
Sex (%)				
Female	64% 16/25	56% 14/25	60% 30/50	NS
Male	36%. 9/25	44% 11/25	40% 20/50	NS
Preoperative BMI (mean, SD)	47.3 ± 6.58 kg/m^2^	45.95 ± 5.85 kg/m^2^	45.99 ± 6.25 kg/m^2^	NS
T2DM	28% 7/25	20% 5/25	24% 12/50	NS
Hypertension	56% 14/25	28% 7/25	42% 21/50	NS
OSAS	24% 6/25	8% 2/25	16%. 8/50	NS

LSG: laparoscopic sleeve gastrectomy; LBSG: laparoscopic banded sleeve gastrectomy; SD: standard deviation; T2DM: type-2 diabetes mellitus; OSAS; obstructive sleep apnea syndrome.

**Table 2:** Differences in BMI at each follow-up up to 4 years.

Follow-up	Group A (LSG *n* = 25)	Group B (LBSG *n* = 25)	*p* value^*∗*^
Preoperative BMI (mean, SD)	47.3 ± 6.58 kg/m^2^	45.95 ± 5.85 kg/m^2^	0.244
3-month BMI (mean, SD)	37.86 ± 5.72 kg/m^2^	37.58 ± 6.21 kg/m^2^	0.869
6-month BMI (mean, SD)	33.64 ± 6.08 kg/m^2^	32.03 ± 5.24 kg/m^2^	0.325
12-month BMI (mean, SD)	29.72 ± 4.40 kg/m^2^	27.42 ± 4.47 kg/m^2^	0.186
36-month BMI (mean, SD)	28.02 ± 4.21 kg/m^2^	24.32 ± 4.54 kg/m^2^	**0.000205**
48-month BMI (mean, SD)	28.80 ± 4.62 kg/m^2^	24.10 ± 4.52 kg/m^2^	**0.00199**

LSG: laparoscopic sleeve gastrectomy; LBSG: laparoscopic banded sleeve gastrectomy. ^*∗*^ANOVA test.

## Data Availability

The datasets generated and/or analyzed during the current study are available from the corresponding author on reasonable request.
